# Long noncoding RNA-based chromatin control of germ cell differentiation: a yeast perspective

**DOI:** 10.1007/s10577-013-9393-5

**Published:** 2013-11-19

**Authors:** Edwige Hiriart, André Verdel

**Affiliations:** 1 Institut National de la Santé et de la Recherche Médicale (INSERM) U823, Grenoble, France; 2 Université Joseph Fourier, Faculté de Médecine, Institut Albert Bonniot, Grenoble, France; 3 Département Différenciation et Transformation Cellulaire, Domaine de la Merci, Institut Albert Bonniot, 38706 La Tronche Cedex, France

**Keywords:** long noncoding RNA, lncRNA, chromatin, gene silencing, germ cell differentiation, sporulation, yeast, *Schizosaccharomyces pombe*, *Saccharomyces cerevisiae*

## Abstract

Germ cell differentiation, the cellular process by which a diploid progenitor cell produces by meiotic divisions haploid cells, is conserved from the unicellular yeasts to mammals. Over the recent years, yeast germ cell differentiation process has proven to be a powerful biological system to identify and study several long noncoding RNAs (lncRNAs) that play a central role in regulating cellular differentiation by acting directly on chromatin. Remarkably, in the well-studied budding yeast *Saccharomyces cerevisiae* and fission yeast *Schizosaccharomyces pombe*, the lncRNA-based chromatin regulations of germ cell differentiation are quite different. In this review, we present an overview of these regulations by focusing on the mechanisms and their respective functions both in *S. cerevisiae* and in *S. pombe*. Part of these lncRNA-based chromatin regulations may be conserved in other eukaryotes and play critical roles either in the context of germ cell differentiation or, more generally, in the development of multicellular organisms.

## Introduction

The widespread implication of nuclear long noncoding RNAs (lncRNAs) in the regulation of gene expression is now established in a broad range of eukaryotes (Mercer and Mattick 2013; Sabin et al. 2013). However, in most instances, our understanding of the mechanisms involving these RNAs and the specific roles played by RNA are largely unknown. Studies conducted in yeast have greatly contributed to our current knowledge of one of the most detailed RNA-based chromatin silencing process, which is the small RNA-mediated formation of heterochromatin, or silent chromatin, in the fission yeast *Schizosaccharomyces pombe* (Lejeune and Allshire 2011; Moazed 2009; Verdel et al. 2009). In this process, small RNAs produced by activation of a conserved pathway, known as RNA interference (RNAi), guide the RNAi effector complex RNA-induced transcriptional silencing (RITS) to chromatin to induce the formation of heterochromatin (Verdel et al. 2004). It is believed that lncRNAs, under synthesis by the RNA polymerase II, serve as RNA platforms to recruit RITS and other chromatin-modifying complexes to chromatin, to initiate the formation of heterochromatin (Moazed 2009; Motamedi et al. 2004; Verdel and Moazed 2005). Similar RNA-based chromatin silencing mechanisms have since been found in other eukaryotes (Verdel et al. 2009). For example, in plants, RNA mediates the deposition of DNA methylation through an RNAi-based mechanism in a process known as RNA-directed DNA methylation (RdDM) (Zhang and Zhu 2011). In animals, such RNAi-mediated chromatin silencing mechanism has been proposed to be acting also at transposons, although direct evidence is still missing (Bourc’his and Voinnet 2010; Castel and Martienssen 2013). These examples indicate that small RNA-guided chromatin modification is probably conserved in a large number of eukaryotes (Castel and Martienssen 2013; Verdel et al. 2009).

Importantly, in addition to the discovery of RNAi-mediated heterochromatin formation in *S. pombe* (Volpe et al. 2002), other RNA-based chromatin silencing mechanisms have recently been found to act both in *S. pombe* and in *Saccharomyces cerevisiae*. In these cases, RNAi and the production of small RNAs do not seem to play a major role in these processes. Moreover, lncRNAs mostly silence the expression of protein-coding genes, and exert important biological functions rather related to the induction or the progression of yeast germ cell differentiation.

Yeast germ cell differentiation, or sporulation, is the critical developmental program that produces from a diploid cell four haploid cells after two rounds of meiotic divisions. The induction of sporulation has been extensively studied in yeast. Entry into sporulation depends both on the environmental conditions and on the mating type status of the yeast. When nitrogen and carbon sources become limiting, yeast can switch to a pseudohyphal growth where cells dramatically change their morphology by elongating and staying attached after cell division. This leads to formation of long filaments of cells that can invade the medium to search for better growth conditions. Upon further nutrient starvation, yeast ceases growth. In the situation where no yeast of opposite mating type is present in its immediate environment, the cell enters a reversible quiescent state, the G0 phase. In the presence of a yeast of opposite mating type, sporulation can be induced. After conjugation and karyogamy, the newly formed diploid zygote can proceed with premeiotic DNA replication followed by two meiotic divisions. During the first meiotic division, homologous chromosomes pair to allow homologous recombination and crossing overs, and later on separate in two different nuclei. The second round of meiosis resembles mitosis in that it leads to separation of sister chromatids. Following the two meiotic divisions, four nuclei mature into spores by forming a thick wall and compacting their genome. Spores are highly resistant to environment stresses. Once the environmental conditions become favorable to growth, the spores germinate and enter a new cycle of vegetative growth. These major events of sporulation are controlled by a series of successive transcriptional waves that have been characterized particularly in the yeasts *S. cerevisiae* and *S. pombe* (Chu et al. 1998; Mata et al. 2002; Primig et al. 2000).

The signaling pathways sensing the presence of nutrients or monitoring the mating-type identity of the yeast that control the induction of sporulation have been described in detail both for *S. cerevisiae* and *S. pombe* in several excellent reviews (Govin and Berger 2009; Harigaya and Yamamoto 2007; Neiman 2011; Otsubo and Yamamoto 2012; van Werven and Amon 2011). In this review, we thus only briefly describe these regulatory aspects of sporulation. Instead, we focus on recent advances made in identifying mechanisms by which lncRNA molecules act on chromatin to regulate sporulation in *S. cerevisiae* and in *S. pombe*. Interestingly, although both yeasts use RNA as key molecules to control germ cell differentiation, the RNA-dependent mechanisms involved are quite different and act at different steps during sporulation.

## RNA-based chromatin silencing mechanisms control the entry into sporulation in *S. cerevisiae*

### Induction of sporulation is a cell-fate decision governed by the tight transcriptional control of IME gene in *S. cerevisiae*

**Fig. 1:**
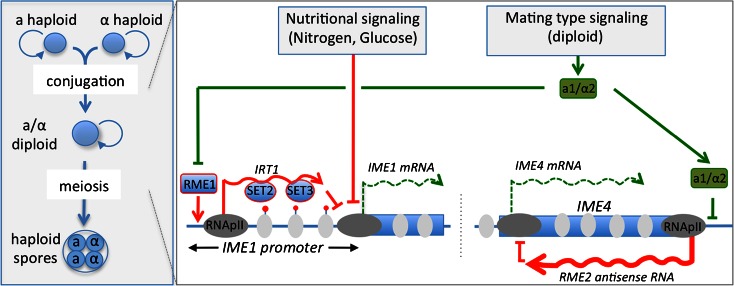
Schematic representation of the lncRNA-based chromatin silencing mechanisms controlling entry into sporulation in *S. cerevisiae. Left box* is a scheme of *S. cerevisiae* sporulation. When environmental conditions are compatible with rapid growth *S. cerevisiae* proliferates by mitotic divisions in either haploid or diploid state. Upon nutrient privation, haploid cells of opposite mating type (a or α) conjugate to form a diploid, which then undergoes premeiotic DNA replication and two rounds of meiotic divisions to produce an *ascus* containing four haploid spores. If the yeast is already diploid it proceeds directly to premeiotic DNA replication. Right box is a schematic overview of the control of sporulation induction at the molecular level that focuses mostly on the RNA-based chromatin silencing mechanisms implicated. *RME1* encodes a transcription factor that binds to, and induces transcription of, *IME1* promoter. Production of Rme1-dependent lncRNA, *IRT1*, promotes the recruitment of histone methyltransferases Set2 and Set3, to the *IME1* promoter and blocks expression of *IME1* presumably by establishing a repressive chromatin environment. Nutrient sensing and mating-type signaling pathways control expression of the master inducer of sporulation, the *IME1* gene. To allow induction of sporulation the nutrient signaling pathways must stop repressing *IME1* expression. In parallel, in haploid cells, the mating-type signaling pathway, by the intermediary of the a1/α2 heterodimeric transcription factor, represses expression of *RME1*. This leads to a block of *IRT1* production and the activation of *IME1* gene expression. Ime1 transcription factor contributes to the activation of a set of genes required for the premeiotic DNA replication. In addition, in haploid cells, another gene important for sporulation, *IME4*, is repressed by the production of antisense RNA, *RME2*. In this case, silencing is imposed by transcriptional interference and this silencing is relieved by the direct binding of the a1/α2 heterodimeric transcription factor to *RME2* promoter, which blocks production of *RME2. Green* is used for pathways promoting sporulation; *Red* for pathways repressing sporulation. *Clear gray circle* nucleosome; *dark gray circle* RNA Polymerase II; *red lollipop* histone post-translational modifications H3K4me and H3K36me. See text for further explanations

Availability of nutritional sources, particularly nitrogen and carbon, are continuously sensed by *S. cerevisiae* to adapt its proliferation status to the growth conditions offered by its environment. Nutrient sensing signaling pathways transmit this information into the nucleus to properly control the induction of the sporulation transcription program. These signaling pathways mostly converge onto the promoter of Inducer of MEiosis 1 (*IME1*) gene. *IME1* gene encodes the master transcription regulator of sporulation, and ectopic expression of *IME1* in diploid cells is sufficient to induce sporulation (Kassir et al. 1988; Smith et al. 1990). When nutrients are not limiting, *S. cerevisiae* undergoes vegetative growth, either as a haploid or a diploid cell, thanks to the repression of *IME1* gene expression by these pathways (Fig. 1) (Neiman 2011; van Werven and Amon 2011). Upon privation of nitrogen and carbon, *IME1* gene repression is relieved. In a haploid cell, the sporulation program must be constitutively inhibited even in the absence of nutrients to avoid the deleterious induction of sporulation in a cell containing only one set of chromosomes as this will lead inevitably to cell death. This block of sporulation is achieved thanks to a mating-type signaling pathway that controls *IME1* gene expression in parallel to the nutrient sensing signaling pathways. When *S. cerevisiae* grows in the haploid state, harboring either the MAT**a** or MATα mating type, *IME1* gene expression is constitutively repressed by the transcription factor Rme1 (Repressor of *IME1*) (Fig. 1) (Covitz and Mitchell 1993; Shimizu et al. 1998). Thus, under nutrient starvation conditions, *IME1* expression is kept silenced until the haploid yeast conjugates with a yeast of opposite mating type to give rise to a diploid cell with a heterozygote *MAT* ***a***/*MATα* mating type. Co-expression of MAT**a** and MATα in the diploid cell leads to the production of the heterodimeric a1/α2 transcription factor that free *IME1* expression from the constitutive silencing by repressing the expression of *RME1* (Covitz et al. 1991; Mitchell and Herskowitz 1986). This event is key to the induction of sporulation. Until recently, the actors and mechanisms involved in the constitutive repression of *IME1* imposed by Rme1 remained poorly understood. Remarkably, at the heart of this silencing mechanism is the production of a lncRNA from the promoter of *IME1*.

### An RNA-based chromatin mechanism silences *IME1* in *cis*

For more than two decades, the exact mode of action of Rme1-mediated repression remained unsolved. Rme1 binds to *IME1* promoter and it efficiently inhibits its transcription (Covitz and Mitchell 1993; Shimizu et al. 1998). Large-scale studies aimed at identifying all RNAs expressed in vegetative cells reported the existence of hundreds of lncRNAs across the entire genome of *S. cerevisiae* (Xu et al. 2009). One of these lncRNAs was found to match the sequence of the *IME1* promoter. Expression of this lncRNA, named *IME1* Regulatory Transcript 1 (*IRT1*), tightly correlates to the growth and differentiation status of the yeast. In cells undergoing vegetative growth, *IRT1* RNA level is relatively high, while upon and during sporulation it goes down (Lardenois et al. 2011; van Werven et al. 2012). RNA accumulation of *IRT1* and *IME1* are thus anticorrelated. This is because Rme1 induces the production of *IRT1*, which blocks in *cis* *IME1* gene expression by different means (van Werven et al. 2012). Production of *IRT1* inhibits the fixation of the transcriptional activator Pog1. In addition, it increases nucleosome occupancy and promotes the recruitment of the histone methyltransferases Set2 and Set3, which have been proposed to establish a repressive chromatin environment repressing *IME1* gene expression. In agreement with such silencing mechanism, Set2 H3K36 methylation mediates the recruitment of the repressive histone deacetylase complex Rpd3S (Fig. 1) (Carrozza et al. 2005; Keogh et al. 2005). Interestingly, a similar silencing mechanism acts at least on one other gene, *SER3*, in *S. cerevisiae*. In this case, transcription of a long noncoding transcript from the promoter region interferes with the binding of transcription factors required for the activation of *SER3* (Martens et al. 2004; Martens et al. 2005). Other genes are also regulated by the transcription of their promoter, which modifies the histone modification pattern of their promoter (Houseley et al. 2008; Pinskaya et al. 2009). Recently, a genome-wide study showed that the recruitment by a variety of lncRNAs of Set3-chromatin-modifying complexes allows a global fine-tuning of gene expression by modulating negatively, but also positively, transcription (Kim et al. 2012). Thus, the lncRNA-based gene silencing mechanism acting at *IME1* promoter not only regulates the critical cell-fate decision to induce sporulation but also acts in a more widespread manner on gene expression control in *S. cerevisiae* (Berretta and Morillon 2009).

### *IME4* gene silencing by antisense transcription

In addition to *IME1*, *IME4* gene is important for the proper induction of sporulation (Shah and Clancy 1992). Depending on the strain background, *IME4* is either required for, or a facilitator of, sporulation (Shah and Clancy 1992). As for *IME1*, *IME4* expression is regulated by a lncRNA, named regulator of meiosis 2 (*RME2*), which, like *IRT1*, acts in *cis* (Fig. 1) (Gelfand et al. 2011; Hongay et al. 2006). However, the mechanism by which *RME2* silences *IME4* is different. *RME2* is an antisense RNA of *IME4* gene (Hongay et al. 2006). During sporulation, expression of *RME2* and *IME4* mRNA are anticorrelated. The production of *RME2* RNA is under the control of a relatively strong promoter compared to *IME4* promoter. Thus, in a haploid cell, heavy transcription of the antisense strand blocks the production of *IME4* sense transcript. The detailed mechanisms involved in silencing *IME4* by *RME2* are not yet understood, but *RME2* has been proposed to block transcription elongation rather than transcription initiation or the binding of transcription factors (Gelfand et al. 2011). At least one other meiotic gene *ZIP2* has its expression regulated in a similar way (Gelfand et al. 2011), indicating that such antisense gene silencing mechanism may apply to several other genes during sporulation. In a heterozygous a/α diploid cell, the a1/α2 heterodimer binds to *RME2* promoter and represses *IME4* antisense transcription to enable transcription of *IME4* and production of Ime4 (Fig. 1). In terms of sporulation control, blocking expression of *RME2*, or of *IRT1*, results in induction of ectopic sporulation in haploid cells or in a/a homozygous diploid cells, but with a lower efficiency than in a/α diploid cells (van Werven et al. 2012). Remarkably, when expression of both *IRT1* and *RME2* lncRNAs is blocked in haploid cells, or in diploid cells with a homozygous mating type, then sporulation takes place with the same kinetics and efficiency than with a/α diploid cells. Conversely, constitutive expression of *IRT1* and *RME2* enable a/α heterozygote diploid cells submitted to sporulation-inducing conditions to enter sporulation. Thus, under nutrient privation, regulation of the production of two lncRNAs is sufficient to dictate whether or not the cell will enter into germ cell differentiation in *S. cerevisiae*.

**Fig. 2:**
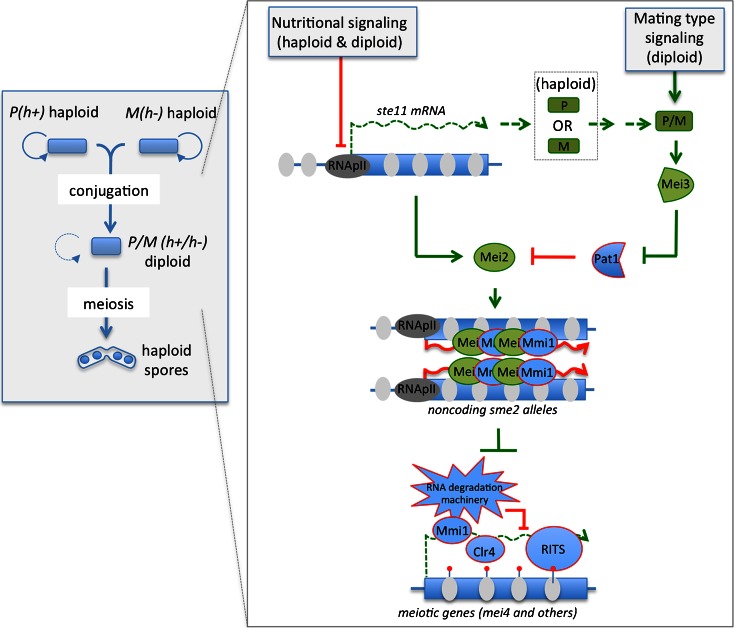
Schematic illustration of the different RNA-based chromatin regulations in connection to sporulation in *S. pombe*, part of which control entry into meiosis. *Left box* is a sketch of *S. pombe* haploid cells undergoing vegetative growth or sporulation. *S. pombe* diploid cells grow in a mitotic manner only on rare occasion, conversely to *S. cerevisiae*. See legend of Fig. 1 for further details. *Right box* depicts the pathways controlling entry into meiosis. At the heart of this control system are the Mei2 RNA-binding protein and the *sme2* long noncoding meiRNAs. Under sporulation-inducing conditions, the nutrient sensing signaling pathways stop repressing *ste11* expression. However, this does not induce sporulation if the yeast is in a *h* + or *h*− haploid state. Ste11 expression will induce expression of P or M factors depending on the haploid state of the yeast, which will promote the conjugation with a partner of opposite mating type to produce a diploid cell. In a diploid state, production of the P and M factors induces expression of Mei3, a nonphosphorylable substrate of Pat1 kinase that inhibits Pat1 activity. Inhibition of Pat1 stabilizes Ste11 and Mei2 proteins, which accumulate in larger quantity. In parallel, production of Ste11 activates transcription of Mei2. A key event for the induction of meiosis is the entry of Mei2 into the nucleus and its accumulation at the noncoding *sme2* gene by binding to the long noncoding meiRNAs. Sequestration of Mmi1 at *sme2* loci is believed to block its repression of specific meiotic genes, including *mei4*, the master regulator activating the middle phase transcription program. During vegetative growth, Mmi1 silences its target genes by degrading their mRNAs thanks to the recruitment of a RNA degradation machinery including the nuclear exosome. In addition, Mmi1 directs the deposition of the heterochromatin mark, H3K9me, by recruiting the histone methyltransferase Clr4 at some of its target meiotic genes. Mmi1 also recruits the RNAi effector complex RITS to these genes. These later aspects may contribute to Mmi1-directed gene silencing and control of sporulation progression. In addition to blocking Mmi1-directed gene silencing by sequestering Mmi1, the long noncoding meiRNAs promote pairing of the two *sme2* loci during meiosis I by an unknown mechanism. *Green* is used for pathways promoting sporulation; *Red* for pathways repressing sporulation. *Red lollipop* histone post-translational modification H3K9me; see text for further explanations

## RNA-based chromatin regulation of entry into meiosis in *S. pombe*

### Induction of sporulation: similarities and distinctions in comparison to *S. cerevisiae*

Induction of sporulation in the fission yeast *S. pombe*, like *S. cerevisiae*, is under the control of signaling pathways monitoring the status of the yeast mating type and sensing nutrients availability (mainly nitrogen) (Harigaya and Yamamoto 2007; Otsubo and Yamamoto 2012). The two yeasts are relatively distant in evolutionary terms (Sipiczki 2000). This translates into molecular mechanisms that are found only in one of them or that have diverged quite significantly. This is the case for the identity of the master regulator as well as for the RNA- and chromatin-based mechanisms regulating sporulation. *S. pombe* master regulator of sporulation is the *ste11* gene, and it does not share homology with *IME1* gene, its *S. cerevisiae* functional counterpart. Nonetheless, a completely different type of RNA-based chromatin regulation plays a critical role at the entry into meiosis in *S. pombe*.

Under nitrogen privation (and poor availability of carbon) *S. pombe* haploid cells of opposite mating type *P* (*or h*+) and *M* (*or h*−) conjugate to form a diploid cell competent for sporulation (Otsubo and Yamamoto 2012). Conversely to *S. cerevisiae*, *S. pombe* proliferates mostly as a haploid cell and forms diploid cells only on rare occasions. Once two *S. pombe* haploid cells have conjugated, the newly formed diploid cell proceeds directly to premeiotic DNA replication followed by two rounds of meiotic divisions to produce four ordered haploid spores (Fig. 2). At the heart of *S. pombe* RNA-based chromatin regulation of sporulation is a lncRNA species produced from the *sme2* genomic locus and named meiRNAs, as well as two RNA-binding proteins called Mei2 and Mmi1. We detail below the mechanisms by which these actors ensure the proper control of both vegetative growth and germ cell development in *S. pombe*.

### Mei2 binding to the long noncoding meiRNAs induces entry into meiosis

Mei2 RNA-binding protein plays a central role in the induction of meiosis (Fig. 2) (Watanabe et al. 1988; Watanabe and Yamamoto 1994). Under sporulation-inducing conditions and after conjugation and karyogamy, Mei2 enters in the nucleus and binds to meiRNAs (Sato et al. 2001; Yamashita et al. 1998). This event is necessary for the diploid cell to proceed through the two meiotic cell divisions and complete sporulation. A serine/threonine protein kinase, Pat1, plays a critical role in regulating sporulation progression notably by phosphorylating Mei2 (Watanabe et al. 1997). Mei2 phosphorylation inhibits its function by at least two means. First, it triggers the degradation of Mei2 by the ubiquitin–proteasome system (Kitamura et al. 2001). Second, it induces Mei2 association with the Rad24 protein, a member of the 14-3-3 phospho-binding protein family (Sato et al. 2002). The association of Rad24 to Mei2 interferes with Mei2 binding with meiRNAs. To allow meiosis induction and progression Pat1 must be inhibited. This is achieved by Mat1-Pm and Mat1-Mm mating type factors that together activate the expression of *mei3* (Van Heeckeren et al. 1998; Willer et al. 1995). Mei3 protein is a pseudosubstrate protein of Pat1 that inhibits Pat1 kinase activity by binding to it (Li and McLeod 1996; McLeod and Beach 1988). Inhibition of Pat1 then leads to a large increase in Mei2 (as well as Ste11) protein level (Kitamura et al. 2001), and to the relocation of Mei2 from the cytoplasm to one specific place in the nucleus called the Mei2 dot (Watanabe et al. 1997).

### Mei2 together with the meiRNA species sequester Mmi1, a key meiotic silencing factor, at *sme2* genomic site

How entry of Mei2 into the nucleus and its localization to the Mei2 dot contribute to meiosis progression remained unclear until the identification of another mechanism critical for the proper control of sporulation. Pioneer work from the group of Masayuki Yamamoto discovered nearly 20 years ago that a gene, named *sme2*, produced the long noncoding meiRNA species, which is absolutely necessary for progression into meiosis I and completion of sporulation in *S. pombe* (Watanabe and Yamamoto 1994). meiRNA species are lncRNAs that can be of two sizes (0.5 or 1.7 kb) and are polyadenylated. Importantly, meiRNA species accumulate at *sme2* locus (Shimada et al. 2003; Yamashita et al. 1998). The mechanism responsible for such retention is not yet known. Sporulation can be induced in *sme2*∆ haploid cells, but after these cells cease vegetative growth, conjugate, and complete premeiotic DNA replication, they arrest upon meiosis I (Watanabe and Yamamoto 1994). Interestingly, some *mei2* mutants show a similar meiotic arrest (Watanabe and Yamamoto 1994). This is probably because meiRNAs and Mei2 act together to sequester a key silencing factor of meiosis, the RNA-binding protein Mmi1 (see below).

Mmi1 belongs to the YTH (YT521 homology) RNA-binding protein family, which has members present in the vast majority of eukaryotes (Stoilov et al. 2002). Quite unexpectedly, Mmi1 was shown to silence the expression of a set of meiotic genes in cells undergoing vegetative growth (Harigaya et al. 2006). Interestingly, Mmi1 silences their gene expression by a post-transcriptional mechanism selectively degrading their mRNAs. Mmi1 itself has no ribonuclease activity. Rather, it directs an RNA degradation machinery to these meiotic mRNAs to efficiently degrade them. Mmi1 insures a selective recognition of these meiotic mRNAs by binding to a consensus hexameric sequence present in several copies in its targets (Hiriart et al. 2012; Yamashita et al. 2012). Once bound to a target mRNA, the RNA degradation machinery recruited by Mmi1 promotes its degradation. The composition of the Mmi1 RNA degradation machinery is only partially known. It includes the nuclear exosome (Harigaya et al. 2006; Hiriart et al. 2012; St-Andre et al. 2010; Sugiyama and Sugioka-Sugiyama 2011; Yamanaka et al. 2010; Zofall et al. 2012). The nuclear exosome is a large multisubunit complex possessing both endo- and exo-ribonucleolytic activities (Chlebowski et al. 2013; Lebreton and Seraphin 2008). Mmi1-mediated recruitment of the nuclear exosome remains to be clarified as it is likely not occurring through a direct binding between Mmi1 and the exosome. Furthermore, it may not involve the exosome co-factor TRAMP, which is known to recruit the exosome at several of its main targets (Lebreton and Seraphin 2008). Instead, Mmi1 associates to proteins related to the control of RNA polyadenylation, the classical polyA polymerase Pla1 and the polyA-binding protein Pab2, and together these proteins have been proposed to recruit the nuclear exosome (Yamanaka et al. 2010). In addition, Mmi1 interacts with a zinc finger protein, Red1, which may also be directly implicated in the exosome recruitment to Mmi1 RNA targets (Sugiyama and Sugioka-Sugiyama 2011). Furthermore, Mmi1 can inhibit the splicing of its target mRNAs, suggesting that Mmi1-directed RNA degradation may act at an early step in gene expression, perhaps co-transcriptionaly (Chen et al. 2011; McPheeters et al. 2009). In agreement with this, Mmi1 binds to chromatin in a transcription-dependent manner (Tashiro et al. 2013; Zofall et al. 2012).

The silencing imposed by Mmi1 RNA surveillance machinery is essential for proper vegetative growth (Harigaya et al. 2006). Among the major targets of Mmi1 RNA surveillance machinery figures the *mei4* gene (Harigaya et al. 2006; Hiriart et al. 2012), which encodes a master transcription factor that activates, directly or indirectly, more than five hundred meiotic genes during sporulation (Mata et al. 2007). Deletion of *mei4* rescues most of the dramatic growth defect observed in *mmi1*∆ cells (Harigaya et al. 2006). Because the deletion of *mei4* mimics the meiotic block observed in *sme2*∆ cells (Kakui et al. 2011; Shimoda et al. 1985; Watanabe and Yamamoto 1994), it is believed that the *sme2*∆ meiotic block is mostly caused by a lack of Mei4 protein due to the constitutive activation of Mmi1 silencing machinery. Interestingly, the meiRNA species itself is a target of Mmi1-directed degradation during vegetative growth (Fig. 2) (Hiriart et al. 2012; Yamashita et al. 2012). This points to an intriguing feedback control where, in mitotically growing cells, the meiRNA species is efficiently degraded by Mmi1 silencing machinery. However, upon induction of sporulation the meiRNAs become somehow immune to Mmi1-directed gene silencing although they still bind to Mmi1. The molecular switch enabling meiRNAs to become potent inhibitors of Mmi1 (to allow meiotic progression) is currently unknown, but Mei2 binding to both the meiRNAs and Mmi1 itself may play an important role in this switch (Harigaya et al. 2006).

### Noncoding RNA-induced chromosome pairing

In addition to its major role in neutralizing Mmi1-directed gene silencing, *sme2* locus exerts another remarkable function linked to chromatin. At the onset of meiosis I, the two *sme2* loci strongly pair regardless of their chromosomal locations (Fig. 2) (Ding et al. 2012a; Ding et al. 2012b). This *sme2* pairing requires production of its long noncoding transcripts, the meiRNAs, but neither Mei2 nor Mmi1-mediated RNA degradation. This represents the first example of a RNA-mediated chromosome pairing. However, its potential conservation in other eukaryotes and the biological significance of this pairing remain to be addressed.

### Mmi1-directed formation of facultative heterochromatin at meiotic genes

In *S. pombe*, formation of constitutive heterochromatin, characterized by histone H3K9 methylation and the presence of HP1-like proteins, takes place at relatively large, noncoding and repeated DNA found mostly at the pericentromeric, subtelomeric, and mating type regions (Grewal 2000). In parallel, heterochromatin marks and other components of heterochromatin were found on few interspersed regions in the genome, including developmentally regulated genes (Cam et al. 2005; Reyes-Turcu and Grewal 2012). The mechanisms responsible for formation of this facultative heterochromatin remained unexplained until the finding that Mmi1 is required for the methylation of histone on its lysine 9 (H3K9me) found at some meiotic genes (Fig. 2) (Hiriart et al. 2012; Tashiro et al. 2013; Zofall et al. 2012). These findings, together with the observation that Mmi1 binds to chromatin in a transcription-dependent manner, suggest that while meiotic mRNAs are under synthesis they may serve as platforms to recruit Mmi1 and the machinery responsible for H3K9 methylation. This is reminiscent to what has been already proposed for lncRNAs emanating from pericentromeric heterochromatin (Moazed 2009; Motamedi et al. 2004). In parallel, Mmi1 recruits the RNAi effector complex RITS to both RNA and chromatin (Fig. 2) (Hiriart et al. 2012). However, conversely to pericentromeric DNA repeats where RNAi plays a dominant role in the deposition of the H3K9me mark and in the overall formation of heterochromatin, RNAi is not required for Mmi1-directed H3K9 methylation at Mmi1 meiotic targets (Zofall et al. 2012). Nonetheless, genetic studies support the idea that RNAi contributes to robust Mmi1-mediated repression of sexual differentiation, possibly by acting mostly at a post-transcriptional level (Hiriart et al. 2012). The role of heterochromatin formation in regulating meiotic gene expression awaits further investigation.

## Conclusion and perspectives

It is estimated that *S. cerevisiae* and *S. pombe* have diverged from a common ancestor at least 300 million years ago (Sipiczki 2000). Thus, they are relatively distant in evolutionary terms. Remarkably, the sporulation processes of the two yeasts are both controlled by lncRNA-based chromatin silencing even though the mechanisms implicated are relatively divergent. Hence, the direct implication and essential function of a RNA-based chromatin regulation of sporulation is a feature conserved between two distant yeasts. Moreover, in both yeasts, more than one RNA-based chromatin silencing mechanism is operating to properly control sporulation. In *S. cerevisiae*, one mechanism imposes gene silencing by producing a lncRNA, within the promoter of a key gene for the induction of sporulation, to serve as a recruiting platform for histone modifying proteins that are believed to establish a repressive chromatin structure. In parallel, production of a noncoding antisense RNA, which may not have any function *per se*, contributes to silence another gene important for sporulation induction. In *S. pombe*, the long noncoding meiRNA species act together with Mei2 RNA-binding protein as a molecular pump or decoy to sequester Mmi1 RNA-binding protein, a key repressor of meiosis, within a specific region of the nucleus. In addition, Mmi1 imposes a potent gene silencing of meiotic genes in vegetative cells, by triggering selective RNA degradation, and inhibiting splicing. Mmi1, by binding to nascent transcripts of protein-coding genes, also induces the formation of facultative heterochromatin that may participate to the silencing of its target genes. Altogether, the variety of RNA-dependent mechanisms implicated in the regulation of yeast germ cell differentiation illustrates the versatility of processes that can be used by RNA to control a key cell-fate decision. An important open question is whether such mechanisms are conserved in other eukaryotes including mammals and whether they exert similar functions.

In animals, the entry of germ cell differentiation has been quite extensively studied, notably in mouse (Saga 2008). The mouse master transcription factor Stra8 controls this developmental switch. Interestingly, *stra8* expression is controlled by the RNA-binding protein Nanos2 (Suzuki and Saga 2008). However, whether an RNA-based chromatin regulation also exists in this case remains unexplored. Yet, given the central role played by this type of process in two distant yeasts and the fact that RNA-based gene silencing plays a central role in controlling Stra8-dependent germ cell differentiation, it would be interesting to test this possibility. The recent findings that PRC1 and PRC2, two chromatin-modifying complexes that associate with lncRNAs (Rinn et al. 2007; Schoeftner et al. 2006), participate in Stra8 silencing (Yokobayashi et al. 2013), further support such possibility.

In animal germ cell differentiation, RNA-based gene silencing exerts an essential function towards transposon. Part of this mechanism is believed to go through long and small RNA-mediated chromatin silencing (Bourc’his and Voinnet 2010). Whether such silencing mechanisms also take place in yeast is still an open question. Indeed, fission yeast transposons have recently been shown to be targets of RNAi and subjected to RNAi-mediated heterochromatin formation in an exosome mutant background (Yamanaka et al. 2013). Whether the existence of potential redundant silencing mechanisms plays any role during germ cell differentiation in *S. pombe* remains to be determined. Ongoing studies on yeast lncRNA-based chromatin regulation in the context of germ cell differentiation should continue to provide important insights into our understanding of how lncRNAs regulate germ cell differentiation and development from yeasts to mammals.

## Abbreviations

IME: Inducer of meiosis
IRT: *IME1* regulatory transcript
lncRNA: Long noncoding RNA
RITS: RNA-induced transcriptional silencing complex
RME: Repressor of *IME1*
RNAi: RNA interference
